# Tumor characteristics and the clinical outcome of invasive lobular carcinoma compared to infiltrating ductal carcinoma in a Chinese population

**DOI:** 10.1186/1477-7819-10-152

**Published:** 2012-07-17

**Authors:** A-Yong Cao, Liang Huang, Jiong Wu, Jin-Song Lu, Guang-Yu Liu, Zhen-Zhou Shen, Zhi-Ming Shao, Gen-Hong Di

**Affiliations:** 1 Breast Cancer Institute, Cancer Centre/Cancer Institute, Shanghai, PR, China; 2 Department of Oncology, Shanghai Medical College, Institutes of Biomedical Science, Fudan University, Shanghai, PR, China; 3 Breast Cancer Institute, Cancer Hospital/Cancer Institute; Department of Oncology, Shanghai Medical College, Fudan University, 270 Dong’an Road, Shanghai, PR 200032, China

**Keywords:** Invasive lobular carcinoma, Tumor characteristics, Clinical outcome

## Abstract

**Background:**

We sought to compare the baseline demographics, standard pathologic factors and long**-**term clinical outcomes between ILC and infiltrating ductal carcinoma (IDC) using a large database.

**Methods:**

Clinicopathologic features, overall survival (OS), and recurrence/metastasis-free survival (RFS) were compared between 2,202 patients with IDC and 215 patients with ILC.

**Results:**

ILC was significantly more likely to be associated with a favorable phenotype, but the incidence of contralateral breast cancer was higher for ILC patients than for IDC patients (8.4% *vs.* 3.9%; *P* =0.001). The frequencies of recurrence/metastasis (*P* = 0.980) and death (*P* = 0.064) were similar among patients with IDC and patients with ILC after adjustment for tumor size and nodal status. The median follow-up was 42.8 months.

**Conclusions:**

Chinese women with ILCs do not have better clinical outcomes than their counterparts with IDC. Management decisions should be based on individual patient and tumor biologic characteristics, and not on lobular histology.

## Background

Lobular carcinoma is the next most common invasive breast cancer histology after ductal carcinoma, accounting for 8% to 14% of all breast cancers [1-4]. Invasive lobular carcinomas (ILC) are believed to be more frequently multicentric and bilateral and may be distinguished from infiltrating ductal carcinoma (IDC) histologically by its cell type and pattern of invasion, as well as by its immunohistochemical profile [5,6]. ILC is also reported to be more difficult to palpate and to visualize with mammography and has a distinctive pattern of metastatic spread [7,8]. Some epidemiologic studies have shown that for unknown reasons, the incidence of this type of breast cancer is increasing, especially among postmenopausal women.

The morphologic features of ILC that are distinct from those of IDC include small, round cells that are bland in appearance and have scant cytoplasm [9]. Because ILC is substantially less common than IDC, knowledge about the clinical outcome of ILC has been based on analysis including relatively small numbers of cases. ILC has been reported to be associated with a poor, similar, or better prognosis than IDC [10-12]. Only limited data have been reported on the biologic features of lobular carcinomas within the context of their clinical outcome. However, some features such as age at diagnosis, tumor size, lymph node status, histological grade, and stage of disease are confirmed to be important prognostic factors for survival in IDC patients [13-15].

We therefore undertook an extensive comparison of ILC and IDC using a large database to provide a more complete and reliable assessment of their biologic phenotypes and clinical behaviors. The present population-based study elucidated the prognosis of women with ILC and IDC with respect to recurrence/metastasis-free survival (RFS) and overall survival (OS) among Chinese women.

## Methods

### Patients and follow-up

This study enrolled 2,417 patients, who were identified histopathologically and treated at the Department of Breast Surgery at Cancer Hospital/Institute, Fudan University (Shanghai, China) during the period from January 1, 1999 to October 1, 2010. These patients were all female and divided into to an ILC group (215 cases) and an IDC group (2,202 cases). Tumors were classified histologically as ILC or IDC only according to the criteria described by World Health Organization (WHO) classification. Each patient was free of distant metastasis at the time of the first diagnosis, and exhibited infiltrative carcinoma. ILC was not further subtyped in these databases, and patients with mixed ILC and IDC were excluded. Also excluded patients with special histologic types (pleomorphic, tubulo-lobular, or other variants such as mucinous, medullary, and *in situ* cancer), and patients with no histological confirmation of the diagnosis, cases identified from autopsy reports only, and patients who did not undergo surgical treatment. Histologic grade and lymphovascular invasion were not analyzed in the present study because in many cases this information was not available, and E cadhaerin test was used in part individuals of our cohort.

All patients were required to undergo a complete physical examination, bilateral mammography, chest radioscopy, ECG, ultrasonography of the breasts, axillary fossa, cervical parts, abdomen and pelvis, and routine blood and biochemical tests before surgery and accompanying adjuvant therapy, according to the standards that were used during surgery. Some patients with early-stage breast cancer were selected for SLNB. The SLN was identified with blue dye (Methylthioninium Chloride Injection, Jiangsu Jumpcan Pharmaceutical Co., Ltd., Shanghai, China) and/or radiocolloid (99 m-Technetium sulfur colloid, CIS US Inc., Bedford, MA, USA). All of the patients at risk of relapse received adjuvant chemotherapy for four to six cycles followed by local radiotherapy (if required) and/or hormonotherapy (if required) according to the standard of therapy at the time of surgery. Follow-up data were collected annually from medical records for breast cancer recurrence, new primary cancers, and death. Personal contact with the patient including routine correspondence or telephone visits was used to follow the patients. The follow-up examinations were performed at the Cancer Hospital of Fudan University every 3 months during the first 2 years, every 6 months during the next 2 years, and once a year thereafter.

### Methods for biological characteristics

The immunohistochemical status of each postoperative paraffin-embedded tumor sample was defined through immunohistochemical staining, including antibodies to estrogen receptor (ER), progesterone receptor (PR), and human epidermal growth factor receptor 2 (HER2/neu). All of the primary monoclonal antibodies were purchased from Dako, Hamburg, Germany. The detailed staining procedures were performed strictly according to the manufacturer’s instructions. Negative controls were obtained by incubating parallel slides without primary antibodies. Sections known to be stained positively in each run served as positive controls. The percentage and intensity score of stained tumor cells (ER, PR, HER2/neu) were determined by at least two independent pathologists. The percentage was interpreted as follows: 0, no staining observed; 1, ≤25% of cells with positive staining; 2, 25% to 50%; 3, 50% to 75%; and 4, 75%. In terms of the intensity score, a score of 0 referred to a negative result, 1 to a weakly positive result, 2 to a moderately positive result and 3 to a strongly positive result. Those two scores were combined and produced a final score. For all these markers except HER2/neu staining, a score of 0 was defined as negative and 1 to 12 as positive, while strong membranous staining scores of 9 to 12 (DAKO score 3+) were defined as positive.

### Statistical analysis

The association of clinicopathologic factors was evaluated using Pearson’s Chi-square or Fisher’s exact tests. The primary clinical outcomes for this study were recurrence/metastasis-free survival (RFS) and overall survival (OS). OS was defined as the time from the first diagnosis of primary breast cancer to death from any cause, and RFS was defined as the time from the same starting point to local recurrence or metastasis. Survival time was calculated from the date of surgery to these endpoints, censoring at the date of last contact and non-breast primaries. The 5-year survival rate was calculated using the Life Tables method. Survival curves were obtained using the Kaplan-Meier method, and the log rank test was used to determine the statistical significance in comparative survival for a variety of patients and tumor characteristics. All of the statistically significant variables observed in univariate analysis were investigated by means of multivariate analysis using the Cox proportional hazards model. All *P* values<0.05 were considered statistically significant. All *P* values are two-sided. The SPSS 15.0 software package (SPSS Inc) was used for statistical analysis.

## Results

### Patient characteristics

Most patients with ILC presented with a palpable mass (191 cases, 84.9%) or nipple discharge (4 cases, 1.9%); 13.2% (20 cases) of the patients only presented with mammographic microcalcifications. We further reviewed the manifestations on mammograms of every ILC patient presenting with a palpable mass and found that 183 cases (85.1%) were accompanied with abnormal radiological changes, including 29 cases (13.5%) with malignant microcalcifications. When the IDC group was concerned, the main primary manifestations at first diagnosis included palpable mass (2,035 cases, 92.4%), microcalcifications detected on mammograms (39 cases, 1.8%), nipple discharge (72 cases, 3.3%), and breast pain, necrosis of the nipple, or other rare symptoms (56 cases, 2.5%). The same evaluations were also performed in those IDC cases presenting with a palpable mass, and a similar positive rate (92.7%, 1,886 cases) of mammographic features was finally observed.

The ILC group comprised 215 cases that were between the ages of 27 years and 85 years; the mean age was 52.5 years. The IDC group was composed of 2,202 cases aged from 23 years to 90 years; the mean age was 51.8 years. Table 1 (shown in supporting information) summarizes the clinical and biologic tumor characteristics according to histologic type. Compared with IDCs, ILCs were much more likely to be bilateral (*P* = 0.001), and ILCs were slightly smaller on average (47.9% smaller than 2 cm) than IDCs (28.3% smaller than 2 cm; *P* = 0.000). Furthermore, ILCs had a stronger association with early breast cancer (TNM = I&II) (*P* <0.001) but presented a relatively higher fraction of disease involving ≥4 axillary nodes (*P* <0.001). More revealingly, the two groups seemed not to be different in terms of the rate of lymph node involvement (48.4% of patients with negative lymph nodes among those with ILCs and 51.3% among those with IDCs, respectively).

**Table 1:** Proportion of invasive lobular and infiltrating ductal histologic types according to baseline characteristics and treatment

**Characteristics**	**Invasive lobular**		**Infiltrating ductal**		***P *value**
	**(*n*** **= 215)**		**(*n*** **= 2,202)**		
	***n***	**%**	***n***	**%**	
*Age (years)*					
≤50	112	52.1	1,138	51.7	0.483
>50	103	47.9	1,064	48.3	
*Bilateral involvement*					
Yes	18	8.4	85	3.9	0.001
No	165	76.7	2,086	94.7	
Unknown	32	14.9	31	1.4	
*Tumor size*					
T ≤ 2	103	47.9	623	28.3	<0.001
2<T ≤ 5	96	44.7	1,277	58.0	
T>5	15	7.0	170	7.7	
Unknown	1	0.5	132	6.0	
*Nodal status*					
0	104	48.4	1,130	51.3	<0.001
1-3	53	24.7	559	25.4	
4-10	26	12.1	347	15.8	
>10	29	13.5	110	5.0	
Unknown	3	1.4	56	2.5	
*TNM*					
I	61	28.4	433	19.7	<0.001
II	91	42.3	1,537	69.8	
III	60	27.9	215	9.8	
Unknown	3	1.4	17	0.8	
*ER status*					
Negative	74	34.4	1,167	53.0	<0.001
Positive	134	62.3	1,028	46.7	
Unknown	7	3.3	7	0.3	
*PR status*					
Negative	78	36.3	1,100	50.0	<0.001
Positive	130	60.5	1,053	47.8	
Unknown	7	3.3	49	2.2	
*HR status*					
Negative	54	25.1	785	35.6	0.004
Positive	154	71.6	1,395	63.4	
Unknown	7	3.3	22	1.0	
*HER2/neu status*					
Negative	170	79.1	1,512	68.7	0.001
Positive	31	14.4	546	24.8	
Unknown	14	6.5	144	6.5	
*Surgery*					
Mastectomy	202	94.0	2,122	96.4	0.079
BCS	13	6.0	80	3.6	
*Chemotherapy*					
Undo	25	11.6	230	10.4	<0.001
MTX included	25	11.6	857	38.9	
Anthracycline included	128	59.5	980	44.5	
Taxane included	32	14.9	30	1.4	
Others	4	1.9	10	0.5	
Unknown	1	0.5	95	4.3	
*Radiation therapy*					
Undo	152	70.7	1,718	78.0	0.001
Do	60	27.9	402	18.3	
Unknown	3	1.4	82	3.7	
*Hormonotherapy*					
Undo	61	28.4	1,216	55.2	<0.001
Do	150	69.8	961	43.6	
Unknown	4	1.9	25	1.1	

BCS, breast-conserving surgery; HR, hormone receptor; IDC, invasive ductal carcinoma; ILC, invasive lobular carcinoma.

Despite the higher rate of bilateral involvement, ILCs had more favorable biologic characteristics (Table 1). The proportion of ER-positive tumors was 62.3% for ILCs, but 46.7% for IDC (*P* < 0.001). PR was expressed in 60.5% of ILCs and in 47.8% of IDCs (*P* <0.001). Regarding HR (ER or PR) status, the ILC group also had a relatively higher HR-positive rate (*P* = 0.004). As for HER2/neu amplification status, ILCs were much more likely to be negative (*P* = 0.001).

With regard to the adjuvant treatment methods, similar proportions of ILC and IDC patients received adjuvant chemotherapy. Likely due to the higher hormone receptor content, adjuvant endocrine therapy was more frequently given to patients with ILC (69.8%) than to those with IDC (43.6%, *P* <0.001). However, the number of patients with ILC who received adjuvant radiotherapy was slightly higher (27.9% in ILC patients *vs.* 18.3% in IDC patients, *P* = 0.001).

The patterns of metastatic dissemination in ILCs and IDCs are shown in Table 2. Lung or pleura, bone, distant node, and liver involvement were frequently observed in both ILCs and IDCs. As metastasis to unusual sites such as the gastrointestinal tract and the ovaries did not appear in ILCs, the difference in the incidence of these metastatic diseases could not be determined. Information on contralateral breast tumors was also available among the subset of 2,354 patients in whom sites of breast cancer distant from the primary site could be assessed. Contralateral breast cancers in this group were more frequent among those with ILC (9.8%) than among those with IDC (3.9%; *P* = 0.001).

**Table 2:** Distant sites of first recurrence/metastasis in this study

**Sites**	**ILC**	**IDC**	***P *value**
	**(n = 183)**	**(n = 2171)**	
Lungs/pleura	5	49	0.811
CNS	1	17	0.726
Ovary	0	3	NA
Gastrointestinal tract	0	1	NA
Nodes	3	53	0.478
Bone	10	107	0.892
Skin	4	33	0.566
Liver	3	37	0.798
Adrenal gland	1	1	0.170

CNS, central nervous system; IDC, invasive ductal carcinoma; ILC, invasive lobular carcinoma; NA, not available.

### Univariate survival analysis

The median follow-up was 42.8 months (range, 5 to 135 months), 41.2 months for the ILC group *vs*. 42.9 months for the IDC group. Contact with 63 patients was lost during the follow-up period.

**Figure 1:**
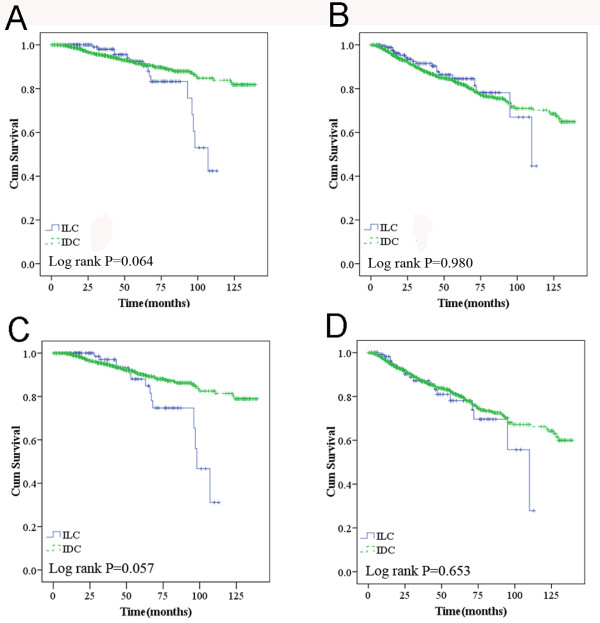
**Overall survival (A) and recurrence/metastasis-free survival (B) according to histologic type; overall survival (C) and recurrence/metastasis-free survival (D) in higher TNM (TNMII&III) stage.** *Adjusted for tumor size (≤2 vs. > 2 cm) and nodal status (positive vs. negative)

In our cohort, women diagnosed with ILC had a similar likelihood of experiencing recurrence/metastasis (*P* = 0.980) and death (*P* = 0.064) as those with IDC. Despite having more favorable biologic characteristics, the 5-year OS and RFS were not better for ILC (83% and 82%) than for IDC (89% and 79%, Figure 1A and B).

**Figure 2:**
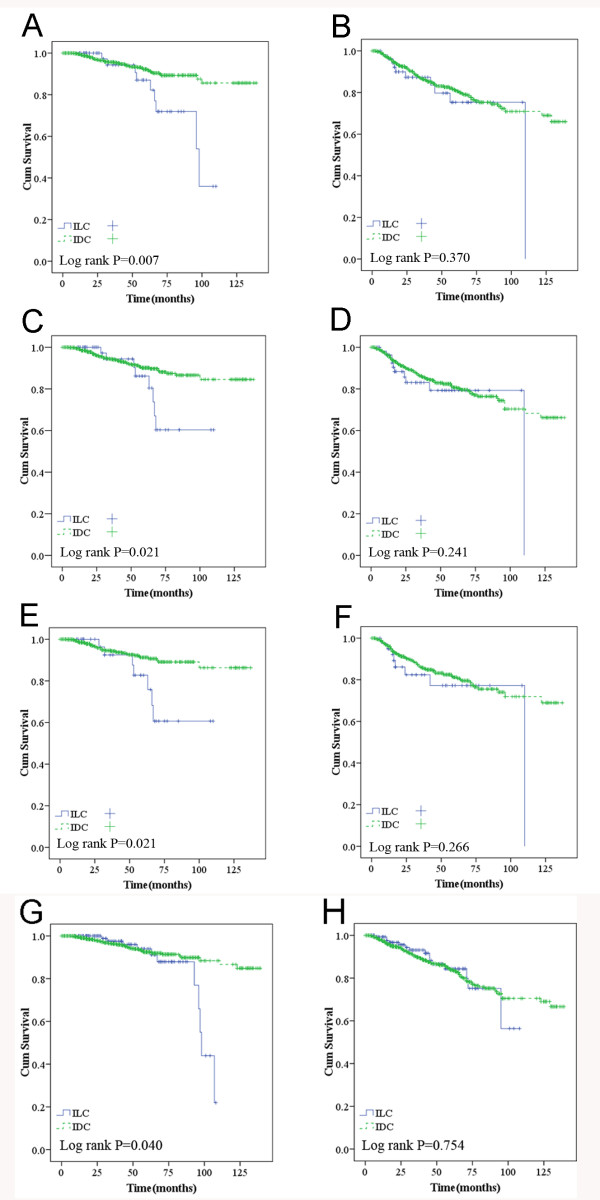
**Overall survival (A) and recurrence/metastasis-free survival (B) according to ER negative; overall survival (C) and recurrence/metastasis-free survival (D) according to PR negative; overall survival (E) and recurrence/metastasis-free survival (F) according to HR negative; overall survival (G) and recurrence/metastasis-free survival (H) in HER2/neu negative.** *Adjusted for tumor size (≤2 *vs.* >2 cm) and lymph node status (positive *vs*. negative).

**Figure 3:**
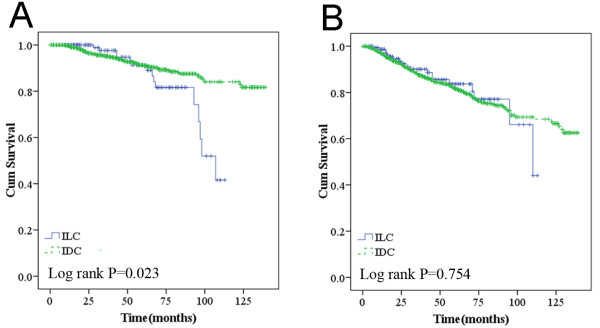
**Overall survival (A) and recurrence/metastasis-free survival (B) in chemotherapy.** *Adjusted for tumor size (≤2 *vs*. >2 cm) and lymph node status (positive *vs*. negative).

When TNM stage was included in the analysis, ILCs with higher TNM stage had a tendency to increase the risk of death compared to those among IDCs (*P* = 0.057 for OS and *P* = 0.653 for RFS) (Figure 2A and B). ILC was also one of the major predictors of worse overall survival in the ER-negative group (Figure 3A, *P* = 0.007), PR-negative group (Figure 3C, *P* = 0.021), HR-negative group (Figure 3E, *P* = 0.021), and HER2/neu-negative group (Figure 3G, *P* = 0.040).

Additionally, ILC women that received chemotherapy had a higher rate of death (*P* = 0.023) compared to their counterparts (Figure 3A).

### Multivariate survival analysis

Multivariate survival analysis was performed to determine whether ILC was an independent prognostic factor for recurrence/metastasis and death. The factors included in these analyses were histological type, age, tumor size, nodal status, TNM stage, ER status, PR status, HR status, HER2/neu status, and adjuvant treatment methods. From among these variables, the factors that independently associated with recurrence/metastasis, as well as with survival in the general population, were as follows: nodal status, TNM stage, ER status, PR status, and HR status (Table 3).

**Table 3:** Cox’s proportional hazards regression models for the general population, ILC and IDC groups

**Variables**	**RFS**		**OS**	
	**RR (95% CI)**	***P *value**	**RR (95% CI)**	***P *value**
*For general*				
Histological type	1.32 (0.82-2.12)	0.248	1.71 (0.91-3.21)	0.098
Age (years) ≤ 50	1.23 (0.94-1.59)	0.131	1.43 (0.75-2.73)	0.284
Tumor size (cm) >2	1.47 (0.90-2.31)	0.124	1.71 (0.73-4.00)	0.215
Nodes positive	1.61 (1.37-1.88)	<0.001	1.40 (1.08-1.81)	0.012
Higher TNM stage	1.56 (1.01-2.24)	0.016	1.78 (1.03-3.07)	0.039
ER positive	0.62 (0.43-0.91)	0.016	0.55 (0.31-1.00)	0.065
PR positive	0.59 (0.40-0.89)	0.011	0.51 (0.28-0.94)	0.031
HR positive	0.54 (0.32-0.91)	0.022	0.46 (0.21-1.02)	0.054
HER2/neu positive	1.21 (0.93-1.58)	0.157	1.24 (0.83-1.85)	0.304
Chemotherapy	0.57 (0.32-1.03)	0.060	0.57 (0.24-1.35)	0.200
Radiotherapy	0.54 (0.40-0.75)	<0.001	1.21 (0.74-2.02)	0.414
Hormonotherapy	0.68 (0.51-0.90)	0.008	0.73 (0.47-1.13)	0.159
*For ILC*				
Age (years) ≤ 50	5.72 (0.83-39.32)	0.076	0.92 (0.81-1.04)	0.182
Tumor size (cm) >2	2.51 (0.61-10.41)	0.204	2.21 (0.97-5.01)	0.058
Nodes positive	2.87 (1.06-7.69)	0.038	5.55 (0.93-33.33)	0.060
Higher TNM stage	4.79 (2.79-11.85)	0.002	3.31 (2.15-9.45)	0.006
ER positive	1.04 (0.175-6.21)	0.964	1.98 (0.15-25.38)	0.600
PR positive	0.52 (0.10-2.58)	0.420	0.28 (0.02-3.24)	0.308
HR positive	0.66 (0.05-9.24)	0.760	0.39 (0.01-20.20)	0.643
HER2/neu positive	1.45 (0.41-5.18)	0.569	0.85 (0.21-3.42)	0.822
Chemotherapy	0.29 (0.02-4.73)	0.383	0.32 (0.12-1.05)	0.060
Radiotherapy	0.86 (0.27-2.78)	0.809	1.22 (0.26-5.72)	0.796
Hormonotherapy	1.38 (0.42-4.54)	0.595	0.90 (0.21-3.76)	0.889
*For IDC*				
Age (years) ≤ 50	1.21 (0.92-1.59)	0.186	1.55 (1.00-2.34)	0.049
Tumor size (cm) >2	1.39 (0.84-2.32)	0.201	2.34 (0.84-6.55)	0.105
Nodes positive	1.62 (1.37-1.91)	<0.001	1.66 (1.27-2.17)	<0.001
Higher TNM stage	1.52 (1.03-2.23)	0.056	1.93 (1.07-3.48)	0.029
ER positive	0.62 (0.42-0.93)	0.021	0.51 (0.27-0.97)	0.040
PR positive	0.60 (0.39-0.91)	0.016	0.47 (0.25-0.91)	0.025
HR positive	0.53 (0.31-0.92)	0.023	0.38 (0.16-0.88)	0.025
HER2/neu positive	1.19 (0.91-1.57)	0.206	1.26 (0.82-1.94)	0.292
Chemotherapy	0.60 (0.33-1.10)	0.099	0.82 (0.33-2.03)	0.662
Radiotherapy	0.51 (0.38-0.69)	<0.001	0.47 (0.30-0.75)	0.002
Hormonotherapy	0.64 (0.47-0.86)	0.004	0.67 (0.42-1.08)	0.101

HR, hormone receptor; IDC, invasive ductal carcinoma; ILC, invasive lobular carcinoma; OS, overall survival; RFS, relapse/metastasis free survival.

In addition, to determine whether traditional prognostic factors for IDC would be of value in patients with ILC, a second set of multivariate analyses of RFS and OS were performed in patients with ILC and IDC, respectively (Table 3). For ILC, only nodal status and TNM stage retained their independent prognostic value, while hormone receptor status remained as a prognostic factor in the IDC group, which is consistent with the findings in the general population.

Once adjustments were made based on tumor size and nodal status, histologic type did not emerge as an important prognostic factor. Thus, the lack of prognostic significance related to ILC *vs.* IDC in univariate analyses is confirmed by the results of the multivariate analyses.

## Discussion

The present retrospective study demonstrated that ILC has distinctive clinicopathologic characteristics compared with IDC in a Chinese population. Largely in agreement with other series, we revealed that ILC in Chinese women was more likely to be smaller in size, of a lower TNM stage, ER or PR positive, and HER2/neu negative. Despite a substantially less aggressive biologic phenotype, recurrence/metastasis and survival were very similar between ILC and IDC patients.

Our database included the cases at an interval of 11 years, and the majority of these cases were treated between 1999 and 2005. The proportion of patients who had breast mastectomy was very high (>95%) in this period. Breast conservation has been increasingly used during the past 5 years in our hospital, and up to 20% of all patients underwent breast-conserving surgery (BCS) in 2011. We found in our cohort that ILCs were treated with BCS as often as IDCs, which was not consistent with other published reports [16-18]. The recommendations against BCS for ILC were based on the consensus that ILC was known to more often be multifocal, multicentric, and bilateral. The clinicians always preferred mastectomy to BCS in the treatment of ILC. However, there was recent evidence that BCS in ILC was not associated with increased local relapse rates at 5 years when compared with mastectomy [19,20]. In the current analysis, a total of 13 ILC patients underwent BCS; we did not identify any recurrence/metastasis or death events at an average follow-up period of 39.0 months. Although in practice, the histologic type appears to play a role in the choice of surgical procedure selected, ILC can indeed be treated with BCS (and radiotherapy) when clear margins can be achieved. In the current study, MRI test was used to evaluate every ILC patient receiving BCS, and no one had received re-excision due to a positive incised margin.

Concerning the pattern of metastatic spread, it seemed that the ILC group of Chinese women was not different from their counterparts with IDC group. ILC as well as IDC were likely to affect the lungs or pleura, bone, and liver. More frequent metastasis to unusual sites such as the vulva and the gastrointestinal tract were previously reported in ILC [21,22], but we could not address this issue because of the limited cases analyzed in our database. We found that the incidence of contralateral breast cancer in women with ILC was nearly double that in women with IDC; this finding could make a compelling case for the use of tamoxifen to prevent contralateral breast cancer in women with lobular primaries.

Most studies have demonstrated that ILC tumors tended to be large [23-25], but 47.9% of ICLs were found not to be in excess of 2 cm compared with only 28.3% of IDCs in our data. We further revealed that a palpable mass was observed in 202 cases (93.9%), and this was the most frequent complaint, which might encourage patients to participate in early screenings and obtain a precise diagnosis when the tumor is smaller. Except for the slightly smaller size of the ILCs, the rate of lymph node involvement was comparable in each group; therefore, many more early-stage breast cancers were presented in the ILC group. To our knowledge, very few previous studies had described these findings.

The current population-based study among Chinese women also definitively validated the findings of some published studies indicating that lobular carcinomas are significantly more likely to be steroid receptor positive than are IDCs [22]. We also evaluated the well-studied growth factor receptors, HER2/neu. No more than 15% of tumors classified as ILC over-expressed HER2/neu; however, 24.8% of IDC patients displayed tumors that overexpressed this receptor. Together these findings suggest that ILC is biologically different from IDC, and has more favorable biologic characteristics.

The RFS and OS curves showed an early advantage for the ILC cohort, but after 4 years, an advantage emerged for the IDC cohort. After adjustment for tumor size and nodal status, there was no prognostic difference between IDC and ILC in the current analysis, which indicated that the more favorable prognostic factors of ILC did not translate into a long-term survival advantage for patients with ILC. We further clarified this important finding through a multivariate analysis in different groups. The favorable prognostic factors such as hormone receptor status identified in the general population and IDC group were not applicable in lobular carcinoma. In spite of the lack of survival differences between ILC and IDC in most large datasets, the univariate adverse prognostic effects of ILC phenotype had appeared to be restricted to women with HR and HER2/neu-negative breast cancer, the majority of whom received adjuvant chemotherapy. We therefore inferred ILC might be associated with more aggressive biologic behavior than IDC in some subgroups and induce a lack of responsiveness to chemotherapy treatment.

Two inherited limitations in this study should be addressed. One potential weakness was the relatively small sample of ILC, so the results in our analysis might not comprehensively account for all the distinct biology and exact prognosis of ILC. The other was that pathologic information for lymphovascular invasion and histologic grade were excluded from the analysis, but these variables could have an effect on survival. Therefore, further studies with larger datasets and more complete pathologic details will be necessary to validate our findings.

## Conclusions

Despite the fact that ILCs are epidemiologically and phenotypically different from IDCs, these patients do not have better clinical outcomes than do patients with IDC. At present, management decisions should be based on individual patient and tumor biologic characteristics, and not on lobular histology.

## Competing interests

There is no any conflict of interest about the study.

## Authors’ contributions

GD carried out the study conception and design. AC was responsible for data collecting and manuscript writing. MH, ZMS and JW participated in the technique support and results analysis. GYL and JSL participated in the design of the study and performed the statistical analysis. ZZS conceived of the study, and participated in its design and coordination and helped to draft the manuscript. All authors read and approved the final manuscript.
